# Helminth Parasite Prevalence in the Endangered Ethiopian Wolf (*Canis simensis*) in Web Valley, Bale Mountains National Park, Ethiopia

**DOI:** 10.1155/2024/6057393

**Published:** 2024-06-28

**Authors:** Girma Ayalew Mengistu, Nigatu Kebede, Fedlu Abdella

**Affiliations:** ^1^ Department of Wildlife Research and Monitoring Ethiopian Wildlife Conservation Authority, Addis Ababa, P.O. Box 386, Ethiopia; ^2^ Akililu lemma Institute of Pathobiology Addis Ababa University, Addis Ababa, P.O. Box 1176, Ethiopia

**Keywords:** Ethiopian wolf, floatation, helminth, parasite, prevalence, sedimentation

## Abstract

Ethiopian wolves (EWs), *Canis simensis*, are the rarest canids in the world and Africa's most endangered carnivore, found in only six isolated habitat fragments in the highlands of Ethiopia. Previous reports on the prevalence of parasites in the EW in Bale Mountains National Park (BMNP) are limited, with little information on their helminth fauna. This study seeks to understand the prevalence of helminth parasites in the EW in BMNP, Ethiopia. In this study, fecal samples were collected from 43 EWs in Web Valley (WV), BMNP, from June to October 2020, and the presence of helminth eggs was assessed using fecal sedimentation and centrifugal floatation methods with microscopy. Forty-two out of 43 fecal samples from wolves (98%) contained eggs from two taxonomic groups of helminths. Eggs from *Capillaria* spp. and *Trichuris vulpis* were found most frequently, followed by *Toxocara canis*, *Diphyllobothrium* spp., *Toxascaris leonina*, and *Capillaria aerophila*. One EW (2%) was recorded for harboring the cestode *Moniezia expansa*. About 9 of the 43 EWs (21%) presented monospecific infection: 9 EWs (21%) harbored 2 parasite species, 9 EWs (21%) hosted 3 parasite species, 11 EWs (26%) had infection involving 4 parasite species, 2 EWs (5%) were infected with 5 parasite species, 1 EW (2%) presented 6 parasite species, 1 EW (2%) harbored 7 parasite species, and 1 EW (2%) was diagnosed without parasite species. Concurrent helminth infection was highly associated with female EW. Megeti 3 was associated with a low level of concurrent helminth infection. The prevalence of helminth parasites found in wolves in the study area suggests that the environment is highly contaminated with intestinal parasites. Regular control of parasite transmission in EW, domestic dogs, and humans in and around BMNP, public education, and further parasite epidemiological studies must be conducted.

## 1. Introduction

Helminth parasites can be found in various ecosystems worldwide, infecting both wild and domestic animals [1, 2]. Helminth eggs can remain viable for varying durations, ranging from 1 to 2 months in crops to several years in fecal sludge and sewage sludge, as well as in soil, fresh water, and sewage [3–5].

Helminths, a diverse class of parasites, have the ability to infect various hosts such as humans, animals (both domestic and wild), and plants [6, 7]. Helminths have been shown to significantly impact animal productivity through various means, such as decreased milk production, slower growth, reduced fertility, and increased vulnerability to other diseases [7, 8].

Carnivores typically serve as definitive hosts, spreading parasite infections by releasing infective eggs or larvae into the environment or infecting intermediate hosts that can be consumed by other hosts [9, 10]. Although some species of helminth parasites have been reported in Ethiopian wolf (EW) in previous studies, the EW has not long been the focus of scientific studies on parasite infections.

Domestic dogs are present throughout most of the known range of EW in the park and at higher densities in the villages and settlements in and around the protected area. The agropastoral community maintains a significant population of domestic dogs (an average of 2.2 dogs per household) in the EW range to protect their livestock from predators, notably spotted hyenas [11, 12].

The presence of a large number of domestic dogs in the habitat of EW is the most immediate threat faced by EW in the Bale Mountains National Park (BMNP) [13, 14]. The situation becomes more complex due to the encroachment of human communities into natural habitats, resulting in the presence of domestic carnivores; some of these are allowed to roam freely [13], which can further endanger the EW and introduce the risk of parasite transmission [14].

As the dog population in the BMNP grows [13], the potential for increased food competition and disease transmission between wolves and dogs also increases. The presence of domestic dogs poses a significant and pressing threat to the EW population that inhabits the highland habitats of the BMNP. This region encompasses crucial habitat for the EW, which is a rare canid species endemic to the Ethiopian highlands and holds the status of Africa's most endangered carnivore [13, 15]. The main conservation challenges in this area include a dense dog population, livestock farming activities, and the prevalence of diseases, all of which directly impact wildlife conservation efforts, especially EW [15]. The EW stands out from other large canids due to its unique feeding behavior, as it primarily feeds on Afro-alpine rodents rather than being a generalist feeder like most canids [16].

BMNP is a home to several species of these Afro-alpine rodents [17], which have also been shown to be favorable intermediate hosts of endoparasites [18, 19]. EWs have also been observed to feed on rock hyraxes, mountain nyala, and livestock calves. Additionally, EWs have been known to scavenge on carcasses of livestock [20]. These feeding behaviors expose the species to potential parasite infections. As a result, EWs are considered vulnerable to parasite transmission due to their interactions with various prey species and the scavenging of livestock carcasses.

The most significant threats responsible for the recent decline in the population of EW are rabies and canine distemper [21–23]. Over the past 5 years, the EW population in the BMNP has experienced a significant decline. The population, which was estimated to be around 300 individuals, has decreased by 30% to only 210 adult individuals. This decline can be attributed to the spread of diseases such as rabies and canine distemper [15]. Following the devastating canine distemper outbreak in 2016, the wolf population in the BMNP experienced a significant decline, leaving behind only 130 adult wolves. This number represents approximately half of the estimated population of 250–300 wolves that would normally inhabit the park under optimal conditions, known as the carrying capacity. The aftermath of this tragic event has left a noticeable void in the wolf population, highlighting the detrimental impact of the outbreak on the natural balance of the ecosystem [24].

Given the slight increase rate observed in the total population in 2017, it was estimated that there were 170 adult and subadult wolves in the BMNP [25]. It remained the largest wolf population in the world, but it was still below its carrying capacity. The wolf population in BMNP is currently in a delicate state, despite its eventual recovery from previous severe outbreaks. Any further mortality or disease occurrence at this critical time could have significant and disproportionate consequences for wolf numbers [25].

The BMNP in Ethiopia is home to three core wolf populations, each residing in optimal habitats. These habitats include the Sanetti Plateau, Web Valley (WV), and Morabawa. The current study focused on the WV habitat, which encompasses approximately 70 km^2^ of prime wolf habitat. This area is known to harbor the highest density of wolves in BMNP [20].

The wolf population in the WV was estimated to be a total of 80 individuals, of which 66 were identified with different earmarks [25]. Although human settlement is slowly encroaching on this wolf habitat, domestic dogs are kept by all households, with up to six dogs per household, for livestock guarding purposes. Households have defined perimeters, but dogs are free to roam the entire WV. Despite the fact that free-roaming and feral dogs (*Canis lupus familiaris*) have been proven by scientific studies to be the main animals responsible for the transmission of rabies and canine distemper to wolves in BMNP [13], to date, no detailed studies on dog parasites that could be transmitted to wolves have been studied or published. Although studies have been conducted on wolf parasites in BMNP only by Jebessa [19] and Van Kesteren et al. [14], currently there is no updated information on the parasite fauna of EW.

The current study was aimed at studying the different helminth species infecting EW in the WV in BMNP and the effects of assumed risk factors for the infection.

## 2. Material and Methods

### 2.1. Study Site

This study was carried out in the WV (7001/N, 39069/E) of BMNP. The valley, located at an elevation of approximately 3500 m, covers an area of approximately 70 km^2^ that provides optimal conditions for the habitation of wolves, characterized by a predominantly flat central region surrounded by steep rocky cliffs to the west and south [20]. The periphery of this region encompasses nine primary villages, alongside several minor homesteads comprising only one to two households, predominantly situated along the valley edges, with gradual expansion encroaching on the central habitat of the wolf population. Households often keep domestic dogs for the purpose of guarding livestock, with an average of 2.2 dogs per household. However, the presence of spotted hyenas, leopards, and occasionally EW can lead to predation of lambs [12, 20].

In the WV, the dominant vegetational disturbances are livestock grazing and mole rat activity, and the primary vegetation is a short herb community, dominated by *Alchemilla* spp. and interspersed with *Helichrysum* and *Artemisia* shrubs. These vast grasslands house a significant population of rodents, with biomass ranging from 2000 to 3000 kg/km^2^ [20]. The wolf population in WV was estimated to be a total of 80 individuals, of which 66 were tagged with different earmarks [25].

The population density of wolves in the Bale region is strongly linked to the presence and abundance of their primary food source, rodents. These rodents thrive in open areas characterized by short vegetation and deep soils. It is in these specific habitats that the wolf population is most concentrated [20, 26, 27].

### 2.2. Study Design

A cross-sectional study was conducted on EWs that were considered the study population in WV within the BMNP.

### 2.3. Preliminary Survey

A preliminary survey and sample collection were conducted in BMNP after permission was obtained, and a support letter was provided from the Ethiopian Wildlife Conservation Authority. As baseline data on current EW packs, domestic dog distribution, settlement, and households in the study area were collected from park office archival documents and Ethiopian Wolf Conservation Program documents. A preliminary survey was conducted in WV prior to the actual data collection period to assess and gather information on the distribution of EW packs.

### 2.4. Collection and Processing of Fecal Samples

EWs were followed on foot and horseback with the help of wolf monitors from the Ethiopian Wolf Conservation Program. Wolves were not tolerant of humans approaching at a close distance, preventing observations from being made while the wolves were defecating; hence, Olympus 8 × 40 Power view binoculars were used.

Whenever a wolf was seen defecating, the top layer of the feces (to avoid contamination from the ground) was collected within minutes of defecation. Age, sex, pack, and identity/ear markings were recorded. Individual wolves were identified by ear markings or tag. Binoculars were used to observe wolves from a distance.

A total of 43 wolves from six known packs were sampled based on the information obtained from the preliminary survey. Out of the 43 sampled wolves, 17 were male, and 15 were female. Of wolves sampled, 14 were adults (classed as ≥ 2 years old), 18 were subadults (≥1 ≤ 2 years old), and 11 were juveniles (≥0.5 ≤ 1 year old) as classified in [14] and Ethiopian Wolf Conservation Program estimates. Juveniles were of unknown sex without ear markings. The adult and subadult wolves had different ear markings.

The 32 wolves with ear marks that were sampled were out of the total of 66 wolves with ear marks in WV. Wolves without ear marks, mainly juveniles, were sampled only once at sight in every wolf pack to avoid resampling.

All samples were collected from target wolf packs based on the information obtained from the preliminary survey. The EW population in WV inhabits the grasslands, and all individual wolves are currently being monitored every week by the wolf monitoring team of the Ethiopian Wolf Conservation Program. The 32 sampled wolves were individually ear-tagged with different visible color marks as part of monitoring activities in the previous rabies vaccination program, and this helped the present study during fecal sampling to exclude animals from resampling.

Fecal samples from each individual wolf were placed in 50 mL bottles and stored in a cooler box on ice until return to the research camp in the BMNP; then, 20 g of a subsection of each sample was preserved in 10% formalin in 40 mL tubes, and the tubes were labeled with weight of the sample, sex, age classification, color of the ear tag and pack of the animal, and ID no.

### 2.5. Sample Transportation and Submission

All tubes containing the samples were properly labeled, kept in a protective container, and transported by a vehicle to be submitted to Addis Ababa University, Aklilu Lemma Institute of Pathobiology (ALIPB), Addis Ababa.

### 2.6. Sample Storage and Processing

In the ALIPB parasitology laboratory, the samples preserved in formalin were stored at room temperature until processed and analyzed. Each sample was processed using sedimentation and centrifugal floatation techniques using salt floatation fluid. This study uses the methods described in [28, 29], and the method description partly reproduces their wording.

### 2.7. Sedimentation Technique

#### 2.7.1. Procedure

Ten grams of feces was weighed on a sensitive balance. Solid feces were ground with mortar and pestle, thoroughly mixed with 100 mL of water, and placed in a beaker. The mixture of feces and water was strained through a tea strainer into another beaker. The mixture was allowed to sit for 30 min, and then, the supernatant was decanted. The water was then added to the previous level, the sediment was resuspended, and the sample was allowed to rest again for 30 min. The supernatant was decanted again, and then, one drop of the sediment was transferred with a pipette to a microscope slide, covered with a coverslip, and examined under a microscope (40x). The result was considered positive when at least one parasite egg was observed.

### 2.8. Centrifugal Floatation Technique

#### 2.8.1. Procedure

A saturated saline solution was prepared in the laboratory by mixing NaCl 500 g in H_2_O 1000 mL, which has specific gravity of 1.2 at saturation.

Three grams of feces was mixed with 40 mL of saline solution, passed through a sieve, and then transferred to 10-mL centrifuge tubes. The tubes were centrifuged at 1200 rpm for 10 min. The test tubes were then placed on a test tube rack, and the solution was added to each tube to form a meniscus, and a coverslip was overlaid. After 15 min, the coverslip was transferred to a glass slide and examined under a microscope (10x and 40x objectives). The result was considered positive when at least one parasite egg was observed.

### 2.9. Identification of Parasite Eggs

Each parasite egg was identified using established structural and morphometric criteria as described in [29–31].

### 2.10. Data Management and Analysis

Raw data on individual animals were entered into a Microsoft Excel spreadsheet program to create a database. The data were then transferred to SPSS version 20 for further analysis. Descriptive statistics were used to summarise the data. The prevalence of infection of each helminth parasite was calculated as the number of animals that harbor any helminth parasite divided by the total number of individuals examined and multiplied by 100 to express in percentage. The chi-square (*χ*^2^) test was used to assess the difference in prevalence of helminth parasites between the variables considered. In all cases, 95% confidence interval (CI) and *p* < 0.05 were set for significance.

Furthermore, the Poisson regression analysis was used to determine the degree of association between different risk factors for the total number of different helminth species detected in the target animals. ArcGIS 10.3.1 software was used to locate the fecal sample of the wolves and the village locations on the map of BMNP.

## 3. Results

### 3.1. Prevalence of Helminth Parasite Infections of the Endangered EW (*Canis simensis*)

Out of the total 43 EWs examined, 97.7% (*n* = 42) were diagnosed as harboring nematode and cestode eggs at varying levels, as indicated in Table 1.

**Figure 1:**
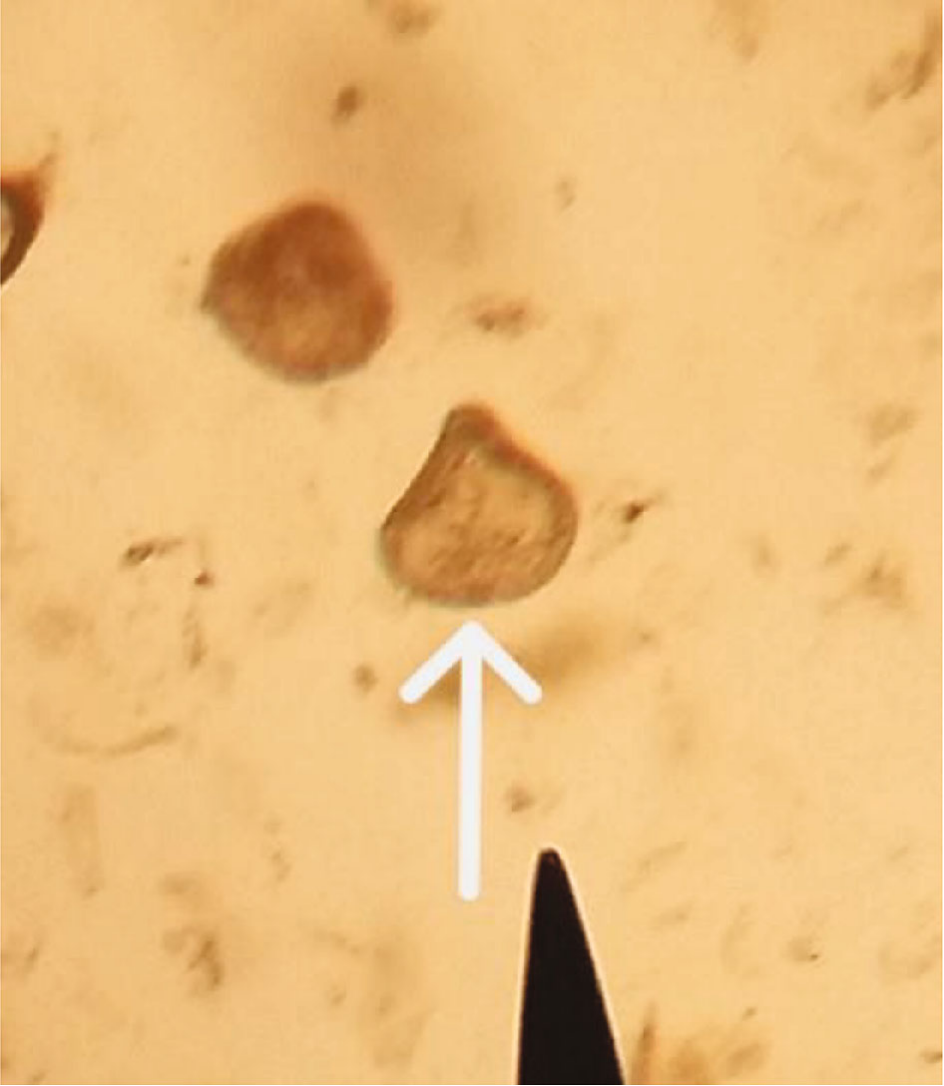
*Moniezia expansa* egg (white arrow) detected from Ethiopian wolf in the study.

Picture of egg of *Moniezia expansa* detected in the wolf fecal sample in the study is shown in Figure 1.

### 3.2. Correlation Between Sex/Age Level/Pack and Concurrent Helminth Parasitism

**Figure 2:**
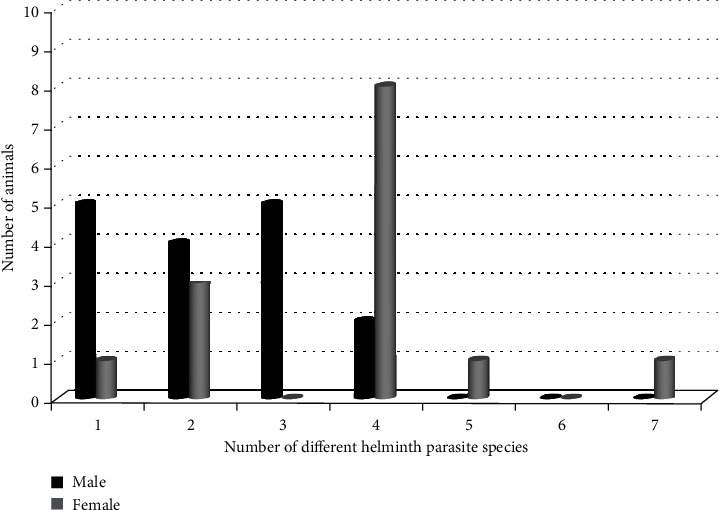
Sex distribution of 32 Ethiopian wolves detected with concurrent helminth parasite species (excluding the 11 juveniles with missing sex values).

**Figure 3:**
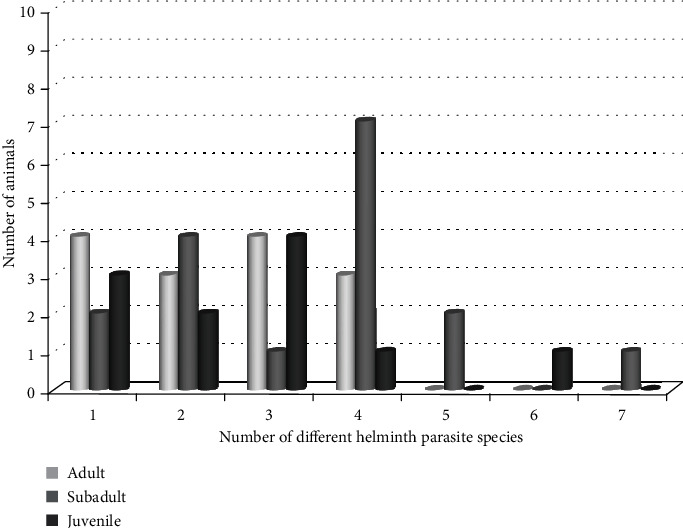
Age distribution of 43 Ethiopian wolves detected with concurrent helminth parasite species (including the 11 juveniles with missing sex values).

A Poisson regression was used to predict the association of concurrent helminth infection with sex, age level, and pack of EW, and the result showed that concurrent helminth infection was highly associated with female EW, but age levels did not show a significant association with concurrent helminth infection (Table 2 and Figures 2 and 3). Similarly, the age-to-sex interaction did not show significant variation. In the sex analysis, the 11 juveniles were excluded for missing sex values but were included in age-level analysis. Similarly, the Habele pack was excluded from the pack analysis for its single value.

**Figure 4:**
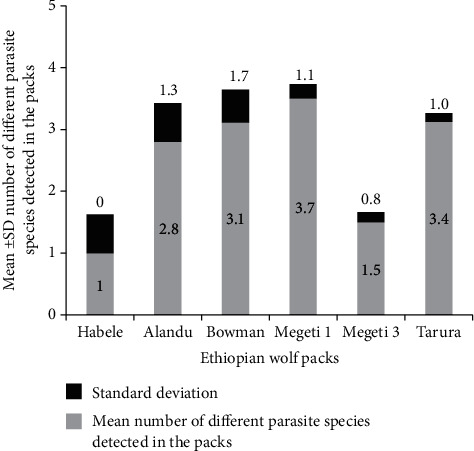
Pack distribution of 43 Ethiopian wolves detected with level of single and concurrent parasite species.

Regarding EW packs, Megeti 3 was associated with low level of concurrent helminth infection, but no significant association was detected between the other EW packs (Table 3 and Figure 4).

## 4. Discussion

Many of the helminth genera discovered in this study have been recorded in closely related canid species [14, 32, 33]. *Toxocara canis*, which was detected in this study, was also commonly found in previous studies in EW in BMNP (25) and recorded most commonly in dogs in different parts of the country [34–37]. Dogs often roam freely in BMNP [13], increasing the chances of transmission to EW. The paratenic hosts, typically rodents, serve as carriers for the infective stage of *Toxocara canis* [38, 39], providing a conducive environment for the parasite's survival and dissemination. In BMNP, wolves are mainly dependent on rodents as their main food source [17], which may contribute to their exposure to *Toxocara canis*.

The occurrence of various species of nematodes that infect EW, as documented in this study, may arise from multiple transmission routes. A potential avenue for infection in EW is the transmission through the ingestion of infected small mammals. Parasites such as *Toxocara* and *Capillaria* are examples of parasites that can be transmitted by this route [14, 40].

As stated in [14], various species of *Capillaria* can infect canids, including *C. aerophila, C. boehmi* [41], *C. plica* [42], and *C. hepatica* [43]. However, it is important to note that none of these infections is expected to result in the presence of eggs in the feces. *C. plica* generally affects the bladder [42], *C. aerophila* is commonly found in the respiratory tract [44], *C. boehmi* primarily affects the nasal cavities and sinuses [41], and *C. hepatica* is known to occur in the liver [45, 46].

This study supports the suggestion of [14] that *Capillaria* eggs could be released into wolf feces after eating prey species infected with this nematode. This is likely because the eggs passed in the feces of infected prey species could then be found in the feces of wolves. *C. hepatica* is frequently found in rodents [36], with a documented infection rate in at least 34 different rodent species [47]. More studies are needed on the parasites of the rodent community of BMNP.

A study by Van Kesteren et al. [14] conducted in BMNP on EW documented helminth eggs of species of *Trichuris*, *Capillaria*, *Toxocara*, Ancylostomatidae, *Hymenolepis*, and Taeniidae, including *Echinococcus granulosus*, but the current study has found three more species of helminth genera, including *Toxascaris leonina*, *Diphyllobothrium* spp., and *Moniezia expansa*.

*T. leonina* may be found in EW as a result of transmission from dogs and other carnivores sharing their habitat. In contrast, *Diphyllobothrium* spp. infection is likely attributed to the consumption of raw fish containing plerocercoid larvae of the parasite. The presence of fish in the diet of EW, as evidenced by their potential access to swamps and rivers within the park, serves as a significant biological tag, supporting the notion that EWs are capable of consuming fish as part of their dietary habits.

In this study, the statistical analysis of the wolf data revealed that wolf age, sex, and pack did not have a significant impact on the presence or absence of the seven investigated helminth species. While this study did not provide information on the abundance or concentration of parasite eggs, it laid the foundation for further investigations to explore the potential impact of parasites on the EW in BMNP.

The relationship between count data and independent variables was examined using the Poisson regression analysis, which identified associations between different risk factors. Specifically, a positive association was found between the sex of EW and concurrent helminth parasite infection, with female wolves being associated with higher rates of concurrent infections.

The observed association of high concurrent infections in female EW could be attributed to several factors. One potential reason for this phenomenon could be related to the physiological stress associated with pregnancy, lactation, and the demands of nurturing offspring. This is supported by research indicating that the immune system of pregnant and lactating animals may be compromised due to the increased metabolic demands and physiological changes during these periods [48, 49]. Additionally, the nurturing role of female wolves in maintaining social harmony within the pack and their involvement in hunting and protecting the pack from potential threats may also contribute to their increased exposure to various infectious agents, leading to a higher prevalence of concurrent infections. Further research into the specific physiological and behavioral factors influencing the susceptibility of female EW to concurrent infections would be valuable in understanding this observed association.

The significantly low rate of concurrent infection in the Megeti 3 wolf pack, as indicated by an odds ratio of 0.444 and a *p* value of 0.012, may be associated with the larger number of collected wolf fecal samples and the diagnosis of no helminth parasite species in one subadult wolf. This observation could be linked to the potential impact of pack size and the distribution of fecal samples on the prevalence of concurrent infections in wolf populations. Larger pack sizes may provide more opportunities for the dilution of infectious agents and parasites, thereby reducing the overall prevalence of concurrent infections within the pack. Additionally, the presence of a subadult wolf with no helminth parasite species could indicate a potential age-related resistance or immunity to certain parasites, contributing to the lower rate of concurrent infections within the Megeti 3 pack.

This study identified the presence of *Moniezia expansa* eggs in a single fecal sample, which is not commonly reported in the existing literature regarding EW. The occurrence of *Moniezia* eggs in the wolf's feces can be attributed to the ingestion of domestic or wild ungulate viscera, where these cestodes are found [50, 51], which explains the presence of *Moniezia* eggs in their feces. This finding may represent pseudoparasitism. Local livestock herders commonly graze sheep, goats, and cattle inside the BMNP [52, 53], and *Moniezia expansa* life cycles could be maintained inside the park. The presence of *Moniezia* eggs in the wolf's feces can serve as a way to prove their predation on sheep, goats, cattle, and wild ungulates, acting as biological tags. This provides evidence of the kind of food ingested by EW and their role as predators in the ecosystem. This study suggests further research to further elucidate the *Moniezia* species present in the Bale Mountains and in EW.

Dogs, accompanied by their agropastoral owners, frequently relocate from one location to another [11, 12]. This constant movement exposes them to new potential parasite infections. Consequently, when the agropastoral people return to areas inhabited by wolves, dogs, now harboring these new parasites, inadvertently transmit them to the wolf population [9, 10, 14].

The presence of zoonotic parasites identified in this study, including *Echinococcus granulosus* discovered in EWs [14], underscores the urgent need for concerted efforts to prioritize zoonotic diseases in Ethiopia. This is particularly crucial given the widespread prevalence of hydatidosis in cattle, sheep, and domestic dogs across Ethiopia [54–57], as well as the prevalence of human hydatidosis in the country. The mean annual incidence rate of human hydatidosis is approximately 2.3 cases per 100,000 per year in Bahir Dar, northern Ethiopia [58], with a prevalence of 0.7% in Hamar, southern Ethiopia [59].

The study by Van Kesteren et al. [14] revealed the presence of various helminth species in EW, including *Capillaria*, *Trichuris*, Ancylostomatidae, *Toxocara*, Taeniidae, and *Hymenolepis*. Among these helminth species, *Trichuris*, Ancylostomatidae, *Toxocara*, and Taeniidae have been reported in domestic dogs residing in Debre Zeit [34], Ambo [35], Hawassa [36], and Jimma [37], which are all approximately 325 km northwest of the BMNP.

Domestic dogs share a close genetic relationship with EW [16] and coexist in the same habitat within the Bale Mountains. Consequently, there is a possibility of parasite transmission between domestic dogs and EW, as they can harbor certain parasites in common.

## 5. Conclusion and Conservation Implications

The present study revealed that there is a wide variety of helminth parasite species in the WV within the BMNP, which is home to endangered and rare EW. Some of these parasites were detected at a high prevalence rate, and concurrent infections involving two or more parasite species were observed to be quite common in this area. Female wolves were particularly associated with this kind of infection.

The prevalence of parasite infection reported in this study may reflect the presence of favorable conditions for environmental contamination and transmission of parasites mainly through the fecal-oral route between the EW and domestic dogs in the study area. To effectively conserve the EW population, it is crucial to incorporate a comprehensive parasite control program for EW and domestic dogs along with the prevention of rabies and canine distemper. This is especially important for dogs that reside in and around the BMNP. By implementing a regular parasite control scheme, we can ensure the overall health and well-being of dogs, reducing the risk of parasite transmission to the EW population. This integrated approach will contribute significantly to the conservation efforts and long-term survival of the EW. Parasite transmission is a more inevitable phenomenon as long as dogs and wolves coexist and share the same environment.

Efforts to mitigate wildlife-dog interactions within the BMNP require a consolidated approach, despite the challenges posed by public perceptions. To ensure the success of EW conservation actions, it becomes imperative to conduct public awareness campaigns that effectively communicate problems arising from dogs and offer strategies to prevent them, such as restricting domestic dog free-roaming within the BMNP. By adopting a more scientifically oriented approach, these conservation initiatives can effectively address concerns surrounding wildlife-dog interactions and promote long-term conservation of the EW population.

The prevalence of *Toxocara canis*, *Capillaria aerophila*, and *Diphyllobothrium* spp. in EW raises concerns about public health due to the potential intertransmissibility between domestic and wild canids, as well as the proximity of wolf habitats to human populations. Domestic dogs have a significant role in the transmission of these parasites to both wildlife and humans, particularly in BMNP. Dogs, as natural definitive hosts for these parasites, excrete eggs into the environment, contributing to the spread of infections to humans and wildlife. The presence of these zoonotic parasites in EW underscores the importance of controlling parasites in domestic and free-roaming dogs to mitigate the risk of transmission to both wildlife and human populations.

More research is needed to evaluate the epidemiology and management strategies pertaining to parasites in the BMNP. The examination of parasites conducted in this study, which highlights the presence of various species in a significant number, underscores the need to understand the impact of parasites on the endangered EW population. Additionally, it emphasizes the urgency to develop and implement interventions aimed at controlling and eradicating parasite transmission and infections.

These findings require a comprehensive understanding of the dynamics of parasite-host interactions and the implementation of effective measures to protect the health and conservation efforts of the EW population in BMNP. This research proposes that conservation initiatives aimed at endangered and threatened EW must incorporate management strategies to mitigate the impact of diseases, particularly those related to parasite infections and transmissions; interactions between dogs, wildlife, and humans; public awareness of disease transmission and wildlife conservation; and the need for comprehensive studies on the epidemiology and control of canine parasites. Failure to address these factors can make conservation efforts ineffective in regions where free-roaming and feral dogs are prevalent.

## Figures and Tables

**Table 1:** Ethiopian wolves tested positive for various helminth species, with the prevalence of these species varying among the wolf population.

**Helminth species**	**Ethiopian wolves (** **N** = 43**)**	
	**Number positive**	**Prevalence (%)**
Nematoda		
*Capillaria* spp.	27	62.8
*Trichuris vulpis*	27	62.8
*Toxocara canis*	23	53.49
*Toxascaris leonine*	7	16.27
*Capillaria aerophila*	6	13.95
Cestoda		
*Diphyllobothrium* spp.	10	23.25
*Moniezia expansa*	1	2.32

**Table 2:** Poisson regression analysis of risk factors for the total number of different helminth species detected in each 32 Ethiopian wolves excluding 11 juveniles for missing sex values for sex analysis and the total 43 Ethiopian wolves including the 11 juveniles for age analysis.

**Risk factor**		**No. of sample examined**	**No. of different helminth species detected per sample (min, max)**	**Odds ratio or Exp (** **B** **) (CI 95%)**	**Wald ** **χ** ^2^	**p** ** value**
Sex	Female	15	(1, 7)	1.763 (1.160, 2.680)	7.045	0.008
	Male	17	(0, 4)	—	—	—

Age	Adult	14	(1, 4)	0.754 (0.494, 1.151)	1.714	0.190
	Sub-adult	18	(0, 7)	—	—	—
	Juvenile	11	(1, 6)	0.818 (0.524, 1.278)	0.779	0.378

Age∗sex (interaction)	—	—	—		1.127	0.288

**Table 3:** Poisson regression analysis of Ethiopian wolf packs as risk factors for the total number of helminth species detected in each 42 Ethiopian wolves excluding a pack (Habele) for its single value.

**Risk factor**	**No. of animals examined**	**No. of different helminth species** **detected per sample (min, max)**	**Odds ratio or Exp (** **B** **) (CI 95%)**	**Wald ** **χ** ^2^	**p** ** value**
Pack					
Alandu	5	(1, 5)	0.830 (0.435, 1.582)	0.322	0.571
Bowman	11	(1, 7)	0.916 (0.553, 1.518)	0.116	0.733
Megeti 1	8	(2, 6)	1.111 (0.661, 1.869)	0.158	0.691
Megeti 3	10	(0, 3)	0.444 (0.236, 0.835)	6.341	0.012
Tarura	8	(1, 4)	—	—	—

## Data Availability

Data are available upon request to the authors.
